# Supplementary material for: The Dual Prey-Inactivation Strategy of Spiders—In-Depth Venomic Analysis of Cupiennius salei
Source: Toxins (Basel). 2019 Mar 19;11(3):167. doi: 10.3390/toxins11030167 (PMC6468893; doi:10.3390/toxins11030167)
Supplement: Supplementary file 1 [file toxins-11-00167-s001.zip › Supplementary Fig S3.1-S3.3/Supplementary Fig S3.1_3.3.pdf]

Supplementary Fig S3.1 – S3.3

Supplementary Fig S3.1

CLUSTAL O(1.2.4) Multiple sequence (cDNA) alignment of signal peptides and propeptides belonging to the SN_19 family

```
Peptide          nucleotide sequence
CsTx-13a         ATGAAAGTTCTCGTTATTTTTGCTGTACTCTCCTTAGTTATTTTCAGCAACTGTTCAGCT    60
CsTx-13b         ATGAAAGTTCTCGTTATTTTTGCTGTACTCTCCTTAGTTATTTTCAGCAACTGTTCAGCT    60
CsTx-10a_S1      ATGAAAGTTCTCGTTATTTTTGCTGTACTCTCCTTAGTTATTTTCAGCAACTGTTCAGCT    60
CsTx-10a_S2      ATGAAAGTTCTCGTTATTTTTGCTGTACTCTCCTTAGTTATTTTCAGCAACTGTTCAGCT    60
CsTx-10b         ATGAAAGTTCTCGTTATTTTTGCTGTACTCTCCTTAGTTATTTTCAGCAACTGTTCAGCT    60
CsTx-11a         ATGAAAGTTCTCGTTATTTTTGCTGTACTCTCCTTAGTTATTTTCAGCAACTGTTCAGCT    60
CsTx-8a_S1       ATGAAAGTTCTCGTTATCTGTGCCGTACTCTTCTTAGCTATTTTCAGCAACAGTTCAGCT    60
CsTx-8a_S2       ATGAAAGTTCTCGTTATCTGTGCCGTACTCTTCTTAGCTATTTTCAGCAACAGTTCAGCT    60
CsTx-9c_S1       ATGAAAGTTCTCGTTATCTGTGCCGTACTCTTCTTAGCTATTTTCAGCAACAGTTCAGCT    60
CsTx-9c_S2       ATGAAAGTTCTCGTTATCTGTGCCGTACTCTTCTTAGCTATTTTCAGCAACAGTTCAGCT    60
CsTx-8b          ATGAAAGTTCTCGTTATCTGTGCCGTACTCTTCTTATCTATTTTCAGCAACAGTTCAGCT    60
CsTx-12a_S1      ATGAAAGTTCTCGTTATCTGTGCTGTACTCTTCTTAACTATTTTCAGCAACAGTTCAGCT    60
CsTx-12a_S2      ATGAAAGTTCTCGTTATCTGTGCTGTACTCTTCTTAACTATTTTCAGCAACAGTTCAGCT    60
CsTx-9b          ATGAAAGTTCTCGTTATCTGTGCTGTACTCTTCTTAACTATTTTCAGCAACAGTTCAGCT    60
CsTx-12b         ATGAAAGTTCTCGTTATCTGTGCCGTACTCTTCTTAGCTATTTTCAGCAACAGTTCAGCT    60
CsTx-9a          ATGAAAGTTCTCGTTATCTGTGCCGTACTCTTCTTAGCTATTTTCAGCAACAGTTCAGCT    60
                 ***************** * *** ******* ****  ************* ********↑identity
CsTx-33a         ATGAAAATTCTCGTTATCTGTGCTGTACTCTTGACAACTATTTGCAGCAAAAGTTCAGCG    60
CsTx-33b         ATGAAAGTTCTCGTTATCTGTGCTGTACTCTTGACAACTATTTGCAGCAAAAGTTCAGCG    60
CsTx-1a_S1       ATGAAAGTTCTCATTATCTCTGCTGTGCTCTTCATAACTATTTTCAGCAACATCTCAGCT    60
CsTx-1a_S3       ATGAAAGTTCTCATTATCTCTGCTGTGCTCTTCATAACTATTTTCAGCAACATCTCAGCT    60
CsTx-1b          ATGAAAGTTCTCATTATCTCTGCTGTGCTCTTCATAACTATTTTCAGCAACATTTCAGCT    60
CsTx-23a         ATGAAGTTTCTGCTCTTTACCGGATTAGTTTTCATTGTGCTCGCAAGCTTGATTGAAGCA    60
CsTx-23b         ATGAAGCTTCTCCTCTTAACCGGATTGTTTTTCATTGTGGTCGTAAGCATGATTGAAGCT    60
all              *****  ****  *  *    *   *  * *         *    ***        *** ↑identity

CsTx-13a         GAAACAGATGAAGATTTCTTCGGAGAAGAAAGTTTTGAAGCTGATGACATAATACCTTTC    120
CsTx-13b         GAAACAGATGAAGATTTCTTCGGAGAAGAAAGTTTTGAAGCTGATGACATAATACCTTTC    120
CsTx-10a_S1      GAAACAGATGAAGATTTCTTCGGAGAAGAAAGTTTTGAAGCTGATGACATAATACCTTTC    120
CsTx-10a_S2      GAAACAGATGAAGATTTCTTCGGAGAAGAAAGTTTTGAAGCTGATGACATAATACCTTTC    120
CsTx-10b         GAAACAGATGAAGATTTCTTCGGAGAAGAAAGTTTTGAAGCTGATGACATAATACCTTTC    120
CsTx-11a         GAAACAGATGAAGATTTCTTCGGAGAAGAAAGTTTTGAAGCTGATGACATAATACCTTTC    120
CsTx-8a_S1       GAAACAGAAGATGATTTCTTGGAAGACGAAAGTTTTCAAGCTGACGACGTAATACCTTTC    120
CsTx-8a_S2       GAAACAGAAGATGATTTCTTGGAAGACGAAAGTTTTCAAGCTGACGACGTAATACCTTTC    120
CsTx-9c_S1       GAAACAGAAGATGATTTCTTGGAAGACGAAAGTTTTCAAGCTGACGACGTAATACCTTTC    120
CsTx-9c_S2       GAAACAGAAGATGATTTCTTGGAAGACGAAAGTTTTCAAGCTGACGACGTAATACCTTTC    120
CsTx-8b          GAAACAGAAGATGATTTCTTGGAAGACGAAAGTTTTCAAGCTGACGACGTAATACCTTTC    120
CsTx-12a_S1      GAAACAGAAGATGATTTCTTGGAAGACGAAAGTTTTGAAGCTGACGACGTAATACCTTTC    120
CsTx-12a_S2      GAAACAGAAGATGATTTCTTGGAAGACGAAAGTTTTGAAGCTGACGACGTAATACCTTTC    120
CsTx-9b          GAAACAGAAGATGATTTCTTGGAAGACGAAAGTTTTGAAGCTGACGACGTAATACCTTTC    120
CsTx-12b         GAAACAGAAGATGATTTCTTGGAAGACGAAAGTTTTGAAGCTGACGACGTAATACCTTTC    120
CsTx-9a          GAAACAGAAGATGATTTCTTGGAAGACGAAAGTTTTGAAGCTGACGACGTAATACCTTTC    120
                 ******** ** ******** * *** ********* ******* *** *********** ↑identity
CsTx-33a         GAAATAGATGAAGACTTCTTGAAAGACGAAAGTTTTGAAGCTGATGGCATAGTACCTTTC    120
CsTx-33b         GAAATAGATGAAGACTTCTTGAAAGACGAAAGTTTTGAAGCTGATGGCATAGTACCTTTC    120
CsTx-1a_S1       GAAATAGAAGATGATTTCTTGGAAGACGAAAGTTTTGAAGCTGAGGACATAATACCTTTC    120
CsTx-1a_S3       GAAATAGAAGATGATTTCTTGGAAGACGAAAGTTTTGAAGCTGAGGACATAATACCTTTC    120
CsTx-1b          GAAATAGAAGATGATTTCTTGGAAGACGAAAGTTTTGAAGCTGAGGACATAATACCTTTC    120
CsTx-23a         GAAGCAGAAAGTGAAAGG------------------------------------------    78
CsTx-23b         GAAGCGGAGAATGAAAGG------------------------------------------    78
all              ***   **    **                                                 ↑identity
```

```
CsTx-13a            ATAGCCAAAGAGCAAGTCAGA         141
CsTx-13b            ATAGCCAAAGAGCAAGTCAGA         141
CsTx-10a_S1         ATAGCCAAAGAGCAAGTCAGA         141
CsTx-10a_S2         ATAGCCAAAGAGCAAGTCAGA         141
CsTx-10b            ATAGCCAAAGAGCAAGTCAGA         141
CsTx-11a            ATAGCCAAAGAGCAAGTCAGA         141
CsTx-8a_S1          CTTGCCAGCGAGCAAGTCAGA         141
CsTx-8a_S2          CTTGCCAGCGAGCAAGTCAGA         141
CsTx-9c_S1          CTTGCCAGCGAGCAAGTCAGA         141
CsTx-9c_S2          CTTGCCAGCGAGCAAGTCAGA         141
CsTx-8b             CTTGCTAGCGAGCAAGTCAGA         141
CsTx-12a_S1         CTTGCCAGGGAACAAGTCAGA         141
CsTx-12a_S2         CTTGCCAGGGAACAAGTCAGA         141
CsTx-9b             CTTGCCAGGGAACAAGTCAGA         141
CsTx-12b            CTTGCCAGGGAACAAGTCAGA         141
CsTx-9a             CTTGCCAGGGAACAAGTCAGA         141
                     * ** *  ** *********       ↑identity
CsTx-33a            TTTGCCAATGAAGAATTCAGA         141
CsTx-33b            TTTGCCAATGAAGAATTCAGA         141
CsTx-1a_S1          TTTGAAAACGAACAAGCCAGA         141
CsTx-1a_S3          TTTGAAAACGAACAAGCCAGA         141
CsTx-1b             TTAGAAAACGAACAAGCCAGA         141
CsTx-23a            ---------------------          78
CsTx-23b            ---------------------          78
```

Percent Identity Matrix - created by Clustal2.1

Nr	1	2	3	4	5	6	7	8
1: CsTx-33a	100.00	99.29	80.85	80.85	80.85	80.85	80.85	80.85
2: CsTx-33b	99.29	100.00	81.56	81.56	81.56	81.56	81.56	81.56
3: CsTx-13a	80.85	81.56	100.00	100.00	100.00	100.00	100.00	100.00
4: CsTx-13b	80.85	81.56	100.00	100.00	100.00	100.00	100.00	100.00
5: CsTx-10a_S1	80.85	81.56	100.00	100.00	100.00	100.00	100.00	100.00
6: CsTx-10a_S2	80.85	81.56	100.00	100.00	100.00	100.00	100.00	100.00
7: CsTx-10b	80.85	81.56	100.00	100.00	100.00	100.00	100.00	100.00
8: CsTx-11a	80.85	81.56	100.00	100.00	100.00	100.00	100.00	100.00
9: CsTx-1a_S1	82.98	83.69	82.27	82.27	82.27	82.27	82.27	82.27
10: CsTx-1a_S3	82.98	83.69	82.27	82.27	82.27	82.27	82.27	82.27
11: CsTx-1b	82.98	83.69	83.69	83.69	83.69	83.69	83.69	83.69
12: CsTx-8a_S1	82.27	82.98	87.23	87.23	87.23	87.23	87.23	87.23
13: CsTx-8a_S2	82.27	82.98	87.23	87.23	87.23	87.23	87.23	87.23
14: CsTx-9c_S1	82.27	82.98	87.23	87.23	87.23	87.23	87.23	87.23
15: CsTx-9c_S2	82.27	82.98	87.23	87.23	87.23	87.23	87.23	87.23
16: CsTx-8b	81.56	82.27	85.82	85.82	85.82	85.82	85.82	85.82
17: CsTx-12a_S1	85.11	85.82	87.23	87.23	87.23	87.23	87.23	87.23
18: CsTx-12a_S2	85.11	85.82	87.23	87.23	87.23	87.23	87.23	87.23
19: CsTx-9b	85.11	85.82	87.23	87.23	87.23	87.23	87.23	87.23
20: CsTx-12b	83.69	84.40	87.23	87.23	87.23	87.23	87.23	87.23
21: CsTx-9a	83.69	84.40	87.23	87.23	87.23	87.23	87.23	87.23
22: CsTx-23a	44.87	44.87	48.72	48.72	48.72	48.72	48.72	48.72
23: CsTx-23b	46.15	46.15	51.28	51.28	51.28	51.28	51.28	51.28

Nr	9	10	11	12	13	14	15	16
1: CsTx-33a	82.98	82.98	82.98	82.27	82.27	82.27	82.27	81.56
2: CsTx-33b	83.69	83.69	83.69	82.98	82.98	82.98	82.98	82.27
3: CsTx-13a	82.27	82.27	83.69	87.23	87.23	87.23	87.23	85.82

**4: CsTx-13b**	82.27	82.27	83.69	87.23	87.23	87.23	87.23	85.82
**5: CsTx-10a_S1**	82.27	82.27	83.69	87.23	87.23	87.23	87.23	85.82
**6: CsTx-10a_S2**	82.27	82.27	83.69	87.23	87.23	87.23	87.23	85.82
**7: CsTx-10b**	82.27	82.27	83.69	87.23	87.23	87.23	87.23	85.82
**8: CsTx-11a**	82.27	82.27	83.69	87.23	87.23	87.23	87.23	85.82
**9: CsTx-1a_S1**	100.00	100.00	98.58	87.23	87.23	87.23	87.23	87.23
**10: CsTx-1a_S3**	100.00	100.00	98.58	87.23	87.23	87.23	87.23	87.23
**11: CsTx-1b**	98.58	98.58	100.00	87.23	87.23	87.23	87.23	87.23
**12: CsTx-8a_S1**	87.23	87.23	87.23	100.00	100.00	100.00	100.00	98.58
**13: CsTx-8a_S2**	87.23	87.23	87.23	100.00	100.00	100.00	100.00	98.58
**14: CsTx-9c_S1**	87.23	87.23	87.23	100.00	100.00	100.00	100.00	98.58
**15: CsTx-9c_S2**	87.23	87.23	87.23	100.00	100.00	100.00	100.00	98.58
**16: CsTx-8b**	87.23	87.23	87.23	98.58	98.58	98.58	98.58	100.00
**17: CsTx-12a_S1**	89.36	89.36	89.36	96.45	96.45	96.45	96.45	95.74
**18: CsTx-12a_S2**	89.36	89.36	89.36	96.45	96.45	96.45	96.45	95.74
**19: CsTx-9b**	89.36	89.36	89.36	96.45	96.45	96.45	96.45	95.74
**20: CsTx-12b**	87.94	87.94	87.94	97.87	97.87	97.87	97.87	96.45
**21: CsTx-9a**	87.94	87.94	87.94	97.87	97.87	97.87	97.87	96.45
**22: CsTx-23a**	50.00	50.00	51.28	51.28	51.28	51.28	51.28	50.00
**23: CsTx-23b**	55.13	55.13	56.41	53.85	53.85	53.85	53.85	52.56

**NR**	**17**	**18**	**19**	**20**	**21**	**22**	**23**
**1: CsTx-33a**	85.11	85.11	85.11	83.69	83.69	44.87	46.15
**2: CsTx-33b**	85.82	85.82	85.82	84.40	84.40	44.87	46.15
**3: CsTx-13a**	87.23	87.23	87.23	87.23	87.23	48.72	51.28
**4: CsTx-13b**	87.23	87.23	87.23	87.23	87.23	48.72	51.28
**5: CsTx-10a_S1**	87.23	87.23	87.23	87.23	87.23	48.72	51.28
**6: CsTx-10a_S2**	87.23	87.23	87.23	87.23	87.23	48.72	51.28
**7: CsTx-10b**	87.23	87.23	87.23	87.23	87.23	48.72	51.28
**8: CsTx-11a**	87.23	87.23	87.23	87.23	87.23	48.72	51.28
**9: CsTx-1a_S1**	89.36	89.36	89.36	87.94	87.94	50.00	55.13
**10: CsTx-1a_S3**	89.36	89.36	89.36	87.94	87.94	50.00	55.13
**11: CsTx-1b**	89.36	89.36	89.36	87.94	87.94	51.28	56.41
**12: CsTx-8a_S1**	96.45	96.45	96.45	97.87	97.87	51.28	53.85
**13: CsTx-8a_S2**	96.45	96.45	96.45	97.87	97.87	51.28	53.85
**14: CsTx-9c_S1**	96.45	96.45	96.45	97.87	97.87	51.28	53.85
**15: CsTx-9c_S2**	96.45	96.45	96.45	97.87	97.87	51.28	53.85
**16: CsTx-8b**	95.74	95.74	95.74	96.45	96.45	50.00	52.56
**17: CsTx-12a_S1**	100.00	100.00	100.00	98.58	98.58	50.00	52.56
**18: CsTx-12a_S2**	100.00	100.00	100.00	98.58	98.58	50.00	52.56
**19: CsTx-9b**	100.00	100.00	100.00	98.58	98.58	50.00	52.56
**20: CsTx-12b**	98.58	98.58	98.58	100.00	100.00	51.28	53.85
**21: CsTx-9a**	98.58	98.58	98.58	100.00	100.00	51.28	53.85
**22: CsTx-23a**	50.00	50.00	50.00	51.28	51.28	100.00	84.62
**23: CsTx-23b**	52.56	52.56	52.56	53.85	53.85	84.62	100.00

Supplementary Fig S3.2

CLUSTAL O(1.2.4) Multiple amino acid sequence alignment of signal peptides and propeptides belonging to the SN_19 family

```
Peptide             Amino acid sequence
CsTx-11a_1          MKVLVIFAVLSLVIFSNCSAETDEDFFGEESFEADDIIPFIAKEQVR   47
CsTx-10b_1          MKVLVIFAVLSLVIFSNCSAETDEDFFGEESFEADDIIPFIAKEQVR   47
CsTx-13b_1          MKVLVIFAVLSLVIFSNCSAETDEDFFGEESFEADDIIPFIAKEQVR   47
CsTx-10a_S2_1       MKVLVIFAVLSLVIFSNCSAETDEDFFGEESFEADDIIPFIAKEQVR   47
CsTx-10a_S1_1       MKVLVIFAVLSLVIFSNCSAETDEDFFGEESFEADDIIPFIAKEQVR   47
CsTx-13a_1          MKVLVIFAVLSLVIFSNCSAETDEDFFGEESFEADDIIPFIAKEQVR   47

CsTx-9c_S1_1        MKVLVICAVLFLAIFSNSSAETEDDFLEDESFQADDVIPFLASEQVR   47
CsTx-9c_S2_1        MKVLVICAVLFLAIFSNSSAETEDDFLEDESFQADDVIPFLASEQVR   47
CsTx-8a_S2_1        MKVLVICAVLFLAIFSNSSAETEDDFLEDESFQADDVIPFLASEQVR   47
CsTx-8a_S1_1        MKVLVICAVLFLAIFSNSSAETEDDFLEDESFQADDVIPFLASEQVR   47
CsTx-8b_1           MKVLVICAVLFLSIFSNSSAETEDDFLEDESFQADDVIPFLASEQVR   47
CsTx-9b_1           MKVLVICAVLFLTIFSNSSAETEDDFLEDESFEADDVIPFLAREQVR   47
CsTx-12a_S1_1       MKVLVICAVLFLTIFSNSSAETEDDFLEDESFEADDVIPFLAREQVR   47
CsTx-12a_S2_1       MKVLVICAVLFLTIFSNSSAETEDDFLEDESFEADDVIPFLAREQVR   47

CsTx-12b_1          MKVLVICAVLFLAIFSNSSAETEDDFLEDESFEADDVIPFLAREQVR   47
CsTx-9a_1           MKVLVICAVLFLAIFSNSSAETEDDFLEDESFEADDVIPFLAREQVR   47
                    ****** *** * ****.****::**: :***:***:***:* ****
CsTx-33a_1          MKILVICAVLLTTICSKSSAEIDEDFLKDESFEADGIVPFFANEEFR   47
CsTx-33b_1          MKVLVICAVLLTTICSKSSAEIDEDFLKDESFEADGIVPFFANEEFR   47
CsTx-23b_1          MKLLLLTGLFFIVVVSMIEAEAENER---------------------   26
CsTx-23a_1          MKFLLFTGLVFIVLASLIEAEAESER---------------------   26
                    **.*:: .:.   : *  .** :.:
```

Percent Identity Matrix - created by Clustal2.1

Nr	1	2	3	4	5	6	7	8
**1: CsTx-23b_1**	100.00	73.08	23.08	23.08	26.92	26.92	26.92	26.92
**2: CsTx-23a_1**	73.08	100.00	23.08	23.08	26.92	26.92	26.92	26.92
**3: CsTx-33a_1**	23.08	23.08	100.00	97.87	61.70	61.70	61.70	61.70
**4: CsTx-33b_1**	23.08	23.08	97.87	100.00	63.83	63.83	63.83	63.83
**5: CsTx-11a_1**	26.92	26.92	61.70	63.83	100.00	100.00	100.00	100.00
**6: CsTx-10b_1**	26.92	26.92	61.70	63.83	100.00	100.00	100.00	100.00
**7: CsTx-13b_1**	26.92	26.92	61.70	63.83	100.00	100.00	100.00	100.00
**8: CsTx-10a_S2_1**	26.92	26.92	61.70	63.83	100.00	100.00	100.00	100.00
**9: CsTx-10a_S1_1**	26.92	26.92	61.70	63.83	100.00	100.00	100.00	100.00
**10: CsTx-13a_1**	26.92	26.92	61.70	63.83	100.00	100.00	100.00	100.00
**11: CsTx-1a_S3_1**	38.46	38.46	63.83	65.96	63.83	63.83	63.83	63.83
**12: CsTx-1a_S1_1**	38.46	38.46	63.83	65.96	63.83	63.83	63.83	63.83
**13: CsTx-1b_1**	38.46	38.46	61.70	63.83	63.83	63.83	63.83	63.83
**14: CsTx-9c_S1_1**	30.77	30.77	61.70	63.83	72.34	72.34	72.34	72.34
**15: CsTx-9c_S2_1**	30.77	30.77	61.70	63.83	72.34	72.34	72.34	72.34
**16: CsTx-8a_S2_1**	30.77	30.77	61.70	63.83	72.34	72.34	72.34	72.34
**17: CsTx-8a_S1_1**	30.77	30.77	61.70	63.83	72.34	72.34	72.34	72.34
**18: CsTx-8b_1**	30.77	30.77	61.70	63.83	72.34	72.34	72.34	72.34
**19: CsTx-9b_1**	30.77	30.77	65.96	68.09	74.47	74.47	74.47	74.47
**20: CsTx-12a_S1_1**	30.77	30.77	65.96	68.09	74.47	74.47	74.47	74.47
**21: CsTx-12a_S2_1**	30.77	30.77	65.96	68.09	74.47	74.47	74.47	74.47
**22: CsTx-12b_1**	30.77	30.77	63.83	65.96	74.47	74.47	74.47	74.47
**23: CsTx-9a_1**	30.77	30.77	63.83	65.96	74.47	74.47	74.47	74.47

Nr	9	10	11	12	13	14	15	16
1: CsTx-23b_1	26.92	26.92	38.46	38.46	38.46	30.77	30.77	30.77
2: CsTx-23a_1	26.92	26.92	38.46	38.46	38.46	30.77	30.77	30.77
3: CsTx-33a_1	61.70	61.70	63.83	63.83	61.70	61.70	61.70	61.70
4: CsTx-33b_1	63.83	63.83	65.96	65.96	63.83	63.83	63.83	63.83
5: CsTx-11a_1	100.00	100.00	63.83	63.83	63.83	72.34	72.34	72.34
6: CsTx-10b_1	100.00	100.00	63.83	63.83	63.83	72.34	72.34	72.34
7: CsTx-13b_1	100.00	100.00	63.83	63.83	63.83	72.34	72.34	72.34
8: CsTx-10a_S2_1	100.00	100.00	63.83	63.83	63.83	72.34	72.34	72.34
9: CsTx-10a_S1_1	100.00	100.00	63.83	63.83	63.83	72.34	72.34	72.34
10: CsTx-13a_1	100.00	100.00	63.83	63.83	63.83	72.34	72.34	72.34
11: CsTx-1a_S3_1	63.83	63.83	100.00	100.00	97.87	72.34	72.34	72.34
12: CsTx-1a_S1_1	63.83	63.83	100.00	100.00	97.87	72.34	72.34	72.34
13: CsTx-1b_1	63.83	63.83	97.87	97.87	100.00	74.47	74.47	74.47
14: CsTx-9c_S1_1	72.34	72.34	72.34	72.34	74.47	100.00	100.00	100.00
15: CsTx-9c_S2_1	72.34	72.34	72.34	72.34	74.47	100.00	100.00	100.00
16: CsTx-8a_S2_1	72.34	72.34	72.34	72.34	74.47	100.00	100.00	100.00
17: CsTx-8a_S1_1	72.34	72.34	72.34	72.34	74.47	100.00	100.00	100.00
18: CsTx-8b_1	72.34	72.34	72.34	72.34	74.47	97.87	97.87	97.87
19: CsTx-9b_1	74.47	74.47	76.60	76.60	78.72	93.62	93.62	93.62
20: CsTx-12a_S1_1	74.47	74.47	76.60	76.60	78.72	93.62	93.62	93.62
21: CsTx-12a_S2_1	74.47	74.47	76.60	76.60	78.72	93.62	93.62	93.62
22: CsTx-12b_1	74.47	74.47	74.47	74.47	76.60	95.74	95.74	95.74
23: CsTx-9a_1	74.47	74.47	74.47	74.47	76.60	95.74	95.74	95.74

Nr	17	18	19	20	21	22	23
1: CsTx-23b_1	30.77	30.77	30.77	30.77	30.77	30.77	30.77
2: CsTx-23a_1	30.77	30.77	30.77	30.77	30.77	30.77	30.77
3: CsTx-33a_1	61.70	61.70	65.96	65.96	65.96	63.83	63.83
4: CsTx-33b_1	63.83	63.83	68.09	68.09	68.09	65.96	65.96
5: CsTx-11a_1	72.34	72.34	74.47	74.47	74.47	74.47	74.47
6: CsTx-10b_1	72.34	72.34	74.47	74.47	74.47	74.47	74.47
7: CsTx-13b_1	72.34	72.34	74.47	74.47	74.47	74.47	74.47
8: CsTx-10a_S2_1	72.34	72.34	74.47	74.47	74.47	74.47	74.47
9: CsTx-10a_S1_1	72.34	72.34	74.47	74.47	74.47	74.47	74.47
10: CsTx-13a_1	72.34	72.34	74.47	74.47	74.47	74.47	74.47
11: CsTx-1a_S3_1	72.34	72.34	76.60	76.60	76.60	74.47	74.47
12: CsTx-1a_S1_1	72.34	72.34	76.60	76.60	76.60	74.47	74.47
13: CsTx-1b_1	74.47	74.47	78.72	78.72	78.72	76.60	76.60
14: CsTx-9c_S1_1	100.00	97.87	93.62	93.62	93.62	95.74	95.74
15: CsTx-9c_S2_1	100.00	97.87	93.62	93.62	93.62	95.74	95.74
16: CsTx-8a_S2_1	100.00	97.87	93.62	93.62	93.62	95.74	95.74
17: CsTx-8a_S1_1	100.00	97.87	93.62	93.62	93.62	95.74	95.74
18: CsTx-8b_1	97.87	100.00	93.62	93.62	93.62	93.62	93.62
19: CsTx-9b_1	93.62	93.62	100.00	100.00	100.00	97.87	97.87
20: CsTx-12a_S1_1	93.62	93.62	100.00	100.00	100.00	97.87	97.87
21: CsTx-12a_S2_1	93.62	93.62	100.00	100.00	100.00	97.87	97.87
22: CsTx-12b_1	95.74	93.62	97.87	97.87	97.87	100.00	100.00
23: CsTx-9a_1	95.74	93.62	97.87	97.87	97.87	100.00	100.00

Supplementary Fig S3.3

CLUSTAL O(1.2.4) multiple amino acid sequence alignment of mature peptides belonging to the SN_19 family

A. One-chain neurotoxins with N-terminal ICK motif, C-terminal a-helical stretch, ±C-terminal amidation

```
SN_19_13
CsTx-1a_S1_1      SCIPKHEECTNDKHNCCRKGLFKLKCQCSTFDDES---GQPTERCACGRPMGHQAIETGLNIFRGLFKGKKKNKKTK(G)
CsTx-1a_S3_1      SCIPKHEECTNDKHNCCRKGLFKLKCQCSTFDDES---GQPTERCACGRPMGHQAIETGLNIFRGLFKGKKKNKKTK(G)
CsTx-1b           SCIPKHEECTNDKHNCCRKGLFKLKCQCSTFDDES---GQPTERCACGRPMGHQAIETGLNIFRGLFKGKKKNKKTK(G)
CsTx-1c_S2        SCIPKHEECTNDKHNCCRRGLFKLKCQCSTFDDES---GQPTERCACGRPMGHQAIETGLNIFRGLFKGKKKNKKTK(G)
CsTx-1c_S1        SCIPKHEECTNDKHNCCRRGLFKLKCQCSTFDDES---GQPTERCACGRPMGHQAIETGLNIFRGLFKGKKKNKKTK(G)
SN_19_14
CsTx-10a_S1   KDKENCIGKHHECTDDRDNCCKGKLFRYQCQCFKVIDGK----KETKRCACVTPLHYKMAEMAVSVFKKMFKN
CsTx-10a_S2   KDKENCIGKHHECTDDRDNCCKGKLFRYQCQCFKVIDGK----KETKRCACVTPLHYKMAEMAVSVFKKMFKN
CsTx-10b      KDKENCIGKHHECTNDRDNCCKGKLFRYQCQCFKVIDGK----KETERCACVTPLHYKMAEMAVSVFKKMFKN
CsTx-11a      KDKENCIGKHHECTNDRDSCCKGKLFRYQCQCFKVIDGK----KETERCACVTPLHYKMAEMAVSVFKKMFKN
CsTx-11b      KDKENCIGKHHECTNDRDSCCKGKLFRYQCQCFKVIDGK----KETKRCACVTPLHYKMAEMAVSVFKKMFKN
```

B. One-chain neurotoxins with N-terminal ICK motif, C-terminal tail, ±C-terminal amidation

```
SN_19_13
CSTX-9a       KDDKNCIPKHHECTNDKKNCCKKGLTKMKCKCFTVADAK---GATSERCACDSSLLQKFGFTGLHIIKGLF
CSTX-9b       KDDKNCIPKHHECTNDKKNCCKKGLTKMKCKCFTVADAK---GATSERCACDSSLLQKFGFTGLHIIKGLF
CSTX-9c_S1    KDDKNCIPKHHECTNDKKNCCKKGLLKLKCQCFTVADEK---GTPSERCACGRPLLHKIAYTGTKMIKGLL
CSTX-9c_S2    KDDKNCIPKHHECTNDKKNCCKKGLLKLKCQCFTVADEK---GTPSERCACGRPLLHKIAYTGTKMIKGLL

CSTX-33a      KDKRNCIPRNQECTIDKRNCCRRGLFKMTCQCMKSNDES---GQPTEKCTCRRPRPIFHLLYKGLLK(G)
CSTX-33b      KDKRNCIPRNQECTIDKRNCCRRGLFKMTCQCMKSNDES---GQPTEKCTCRRPRPIFHLLYKGLLK(G)
SN_19_06
CSTX-23a          ACIEYELECTENKDNCCS----DYKCQCYRRFVKG---VDKGPSCFCLEKDVKYKPL
CSTX-23b          ACIGFELECTKDKKNCCS----DYKCQCYRRFVKG---VDKGQKCWCLDKDVKYKPL
```

C. Two-chain neurotoxins with N-terminal ICK motif, C-terminal a-helical stretch, posttranslational removement, C-terminal amidation

```
SN_19_12
CSTX-13a         SDCTLRNHDCTDDRHSCCRSKMFKDVCTCFYPSQRSETARAKKELCTCQQPKHLKYIEKGLQKAKDYAT(G)
CSTX-13b         SDCTLRNHDCTDDRHSCCRSKMFKDVCTCFYPSQRSETDRAKKELCTCQQPKHLKYIEKGLQKAKDYAT(G)
CSTX-8a_S2       SDCTLRNHDCTDDRHSCCRSKMFKDVCKCFYPSQRSETDRAKKELCTCQQPKHLKYIEKGLQKAKDYAT(G)
CSTX-8a_S1       SDCTLRNHDCTDDRHSCCRSKMFKDVCKCFYPSQRSETDRAKKELCTCQQPKHLKYIEKGLQKAKDYAT(G)
CSTX-8b          SDCTLRNHDCTDDRHSCCRSKMFKDVCKCFYPSQRSETDRAKKELCTCQQPKHLKYIEKGLQKAKDYAT(G)
CSTX-12a_S1      SDCTLRNHDCTDDRHSCCRSKMFKDVCKCFYPSQRSDTARAKKELCTCQQDKHLKFIEKGLQKAKVLVA(G)
CSTX-12a_S2      SDCTLRNHDCTDDRHSCCRSKMFKDVCKCFYPSQRSDTARAKKELCTCQQDKHLKFIEKGLQKAKVLVA(G)
CSTX-12b         SDCTLRNHDCTDDRHSCCRSKMFKDVCKCFYPSQRSDTARAKKELCTCQQDKHLKYIEKGLQKAKVLVA(G)
```

Percent Identity Matrix - created by Clustal2.1

Nr	1	2	3	4	5	6	7	8
1: CsTx-23a	100.00	82.00	24.00	24.00	24.00	24.00	24.00	26.00
2: CsTx-23b	82.00	100.00	26.00	26.00	26.00	26.00	26.00	28.00
3: CsTx-8a_S1	24.00	26.00	100.00	100.00	100.00	97.14	98.57	88.57
4: CsTx-8a_S2	24.00	26.00	100.00	100.00	100.00	97.14	98.57	88.57
5: CsTx-8b	24.00	26.00	100.00	100.00	100.00	97.14	98.57	88.57
6: CsTx-13a	24.00	26.00	97.14	97.14	97.14	100.00	98.57	88.57
7: CsTx-13b	24.00	26.00	98.57	98.57	98.57	98.57	100.00	87.14
8: CsTx-12a_S1	26.00	28.00	88.57	88.57	88.57	88.57	87.14	100.00
9: CsTx-12a_S2	26.00	28.00	88.57	88.57	88.57	88.57	87.14	100.00
10: CsTx-12b	26.00	28.00	90.00	90.00	90.00	90.00	88.57	98.57
11: CsTx-10a_S1	34.69	36.73	31.82	31.82	31.82	31.82	31.82	30.30
12: CsTx-10a_S2	34.69	36.73	31.82	31.82	31.82	31.82	31.82	30.30
13: CsTx-10b	34.69	36.73	31.82	31.82	31.82	31.82	31.82	30.30
14: CsTx-11a	32.65	34.69	33.33	33.33	33.33	33.33	33.33	31.82
15: CsTx-33a	34.00	38.00	33.87	33.87	33.87	33.87	33.87	33.87
16: CsTx-33b	34.00	38.00	33.87	33.87	33.87	33.87	33.87	33.87
17: CsTx-1a-S1	32.00	34.00	33.33	33.33	33.33	33.33	33.33	33.33
18: CsTx-1a-S3	32.00	34.00	33.33	33.33	33.33	33.33	33.33	33.33
19: CsTx-1b	32.00	34.00	33.33	33.33	33.33	33.33	33.33	33.33
20: CsTx-1c-S1	32.00	34.00	33.33	33.33	33.33	33.33	33.33	33.33
21: CsTx-1c-S2	32.00	34.00	33.33	33.33	33.33	33.33	33.33	33.33
22: CsTx-9a	34.00	38.00	30.77	30.77	30.77	29.23	29.23	33.85
23: CsTx-9b	34.00	38.00	30.77	30.77	30.77	29.23	29.23	33.85
24: CsTx-9c_S1	36.00	40.00	27.69	27.69	27.69	27.69	27.69	27.69
25: CsTx-9c_S2	36.00	40.00	27.69	27.69	27.69	27.69	27.69	27.69

Nr	9	10	11	12	13	14	15	16
1: CsTx-23a	26.00	26.00	34.69	34.69	34.69	32.65	34.00	34.00
2: CsTx-23b	28.00	28.00	36.73	36.73	36.73	34.69	38.00	38.00
3: CsTx-8a_S1	88.57	90.00	31.82	31.82	31.82	33.33	33.87	33.87
4: CsTx-8a_S2	88.57	90.00	31.82	31.82	31.82	33.33	33.87	33.87
5: CsTx-8b	88.57	90.00	31.82	31.82	31.82	33.33	33.87	33.87
6: CsTx-13a	88.57	90.00	31.82	31.82	31.82	33.33	33.87	33.87
7: CsTx-13b	87.14	88.57	31.82	31.82	31.82	33.33	33.87	33.87
8: CsTx-12a_S1	100.00	98.57	30.30	30.30	30.30	31.82	33.87	33.87
9: CsTx-12a_S2	100.00	98.57	30.30	30.30	30.30	31.82	33.87	33.87
10: CsTx-12b	98.57	100.00	30.30	30.30	30.30	31.82	33.87	33.87
11: CsTx-10a_S1	30.30	30.30	100.00	100.00	97.10	95.65	40.62	40.62
12: CsTx-10a_S2	30.30	30.30	100.00	100.00	97.10	95.65	40.62	40.62
13: CsTx-10b	30.30	30.30	97.10	97.10	100.00	98.55	42.19	42.19
14: CsTx-11a	31.82	31.82	95.65	95.65	98.55	100.00	40.62	40.62
15: CsTx-33a	33.87	33.87	40.62	40.62	42.19	40.62	100.00	100.00
16: CsTx-33b	33.87	33.87	40.62	40.62	42.19	40.62	100.00	100.00
17: CsTx-1a-S1	33.33	33.33	41.54	41.54	44.62	43.08	57.38	57.38
18: CsTx-1a-S3	33.33	33.33	41.54	41.54	44.62	43.08	57.38	57.38
19: CsTx-1b	33.33	33.33	41.54	41.54	44.62	43.08	57.38	57.38
20: CsTx-1c-S1	33.33	33.33	41.54	41.54	44.62	43.08	59.02	59.02
21: CsTx-1c-S2	33.33	33.33	41.54	41.54	44.62	43.08	59.02	59.02
22: CsTx-9a	33.85	32.31	46.27	46.27	49.25	47.76	44.44	44.44
23: CsTx-9b	33.85	32.31	46.27	46.27	49.25	47.76	44.44	44.44
24: CsTx-9c_S1	27.69	27.69	49.25	49.25	52.24	50.75	52.38	52.38
25: CsTx-9c_S2	27.69	27.69	49.25	49.25	52.24	50.75	52.38	52.38

Nr	17	18	19	20	21	22	23	24	25
1: CsTx-23a	32.00	32.00	32.00	32.00	32.00	34.00	34.00	36.00	36.00
2: CsTx-23b	34.00	34.00	34.00	34.00	34.00	38.00	38.00	40.00	40.00
3: CsTx-8a_S1	33.33	33.33	33.33	33.33	33.33	30.77	30.77	27.69	27.69
4: CsTx-8a_S2	33.33	33.33	33.33	33.33	33.33	30.77	30.77	27.69	27.69
5: CsTx-8b	33.33	33.33	33.33	33.33	33.33	30.77	30.77	27.69	27.69
6: CsTx-13a	33.33	33.33	33.33	33.33	33.33	29.23	29.23	27.69	27.69
7: CsTx-13b	33.33	33.33	33.33	33.33	33.33	29.23	29.23	27.69	27.69
8: CsTx-12a_S1	33.33	33.33	33.33	33.33	33.33	33.85	33.85	27.69	27.69
9: CsTx-12a_S2	33.33	33.33	33.33	33.33	33.33	33.85	33.85	27.69	27.69
10: CsTx-12b	33.33	33.33	33.33	33.33	33.33	32.31	32.31	27.69	27.69
11: CsTx-10a_S1	41.54	41.54	41.54	41.54	41.54	46.27	46.27	49.25	49.25
12: CsTx-10a_S2	41.54	41.54	41.54	41.54	41.54	46.27	46.27	49.25	49.25
13: CsTx-10b	44.62	44.62	44.62	44.62	44.62	49.25	49.25	52.24	52.24
14: CsTx-11a	43.08	43.08	43.08	43.08	43.08	47.76	47.76	50.75	50.75
15: CsTx-33a	57.38	57.38	57.38	59.02	59.02	44.44	44.44	52.38	52.38
16: CsTx-33b	57.38	57.38	57.38	59.02	59.02	44.44	44.44	52.38	52.38
17: CsTx-1a-S1	100.00	100.00	100.00	98.67	98.67	56.25	56.25	64.06	64.06
18: CsTx-1a-S3	100.00	100.00	100.00	98.67	98.67	56.25	56.25	64.06	64.06
19: CsTx-1b	100.00	100.00	100.00	98.67	98.67	56.25	56.25	64.06	64.06
20: CsTx-1c-S1	98.67	98.67	98.67	100.00	100.00	54.69	54.69	62.50	62.50
21: CsTx-1c-S2	98.67	98.67	98.67	100.00	100.00	54.69	54.69	62.50	62.50
22: CsTx-9a	56.25	56.25	56.25	54.69	54.69	100.00	100.00	75.00	75.00
23: CsTx-9b	56.25	56.25	56.25	54.69	54.69	100.00	100.00	75.00	75.00
24: CsTx-9c_S1	64.06	64.06	64.06	62.50	62.50	75.00	75.00	100.00	100.00
25: CsTx-9c_S2	64.06	64.06	64.06	62.50	62.50	75.00	75.00	100.00	100.00
